# Developing a rapid and highly efficient cowpea regeneration, transformation and genome editing system using embryonic axis explants

**DOI:** 10.1111/tpj.15202

**Published:** 2021-03-16

**Authors:** Ping Che, Shujun Chang, Marissa K. Simon, Zhifen Zhang, Ahmed Shaharyar, Jesse Ourada, Dennis O’Neill, Mijael Torres‐Mendoza, Yinping Guo, Kathleen M. Marasigan, Jean‐Philippe Vielle‐Calzada, Peggy Ozias‐Akins, Marc C. Albertsen, Todd J. Jones

**Affiliations:** ^1^ Corteva Agriscience Johnston Iowa 50131 USA; ^2^ Department of Horticulture and Institute of Plant Breeding, Genetics & Genomics University of Georgia Tifton Campus Tifton GA 31973 USA; ^3^ Group of Reproductive Development and Apomixis, UGA Laboratorio Nacional de Genómica para la Biodiversidad CINVESTAV Irapuato Guanajuato 36821 México; ^4^ Present address: Benson Hill Biosystems 1100 Corporate Square Dr. Suite 150 St. Louis MO 63132 USA

**Keywords:** cowpea transformation, embryonic axis, cotyledonary node (cot‐node), shoot organogenesis, spectinomycin, *Agrobacterium*, CRISPR/Cas

## Abstract

Cowpea (*Vigna unguiculata* (L.) Walp.) is one of the most important legume crops planted worldwide, but despite decades of effort, cowpea transformation is still challenging due to inefficient *Agrobacterium*‐mediated transfer DNA delivery, transgenic selection and *in vitro* shoot regeneration. Here, we report a highly efficient transformation system using embryonic axis explants isolated from imbibed mature seeds. We found that removal of the shoot apical meristem from the explants stimulated direct multiple shoot organogenesis from the cotyledonary node tissue. The application of a previously reported ternary transformation vector system provided efficient *Agrobacterium*‐mediated gene delivery, while the utilization of *spcN* as selectable marker enabled more robust transgenic selection, plant recovery and transgenic plant generation without escapes and chimera formation. Transgenic cowpea plantlets developed exclusively from the cotyledonary nodes at frequencies of 4% to 37% across a wide range of cowpea genotypes. CRISPR/Cas‐mediated gene editing was successfully demonstrated. The transformation principles established here could also be applied to other legumes to increase transformation efficiencies.

## Significance Statement

The tissue culture and transformation technology developed herein represents a significant advance in *Agrobacterium*‐mediated transformation and CRISPR/Cas‐mediated genome editing of an important orphan crop, cowpea (*Vigna unguiculata* (L.) Walp.). The principles established in this study have the potential to improve the transformation and editing efficiencies not only for cowpea, but also for other legume species, such as soybean (*Glycine max*) and common bean (*Phaseolus vulgaris*).

## INTRODUCTION

Domesticated in Africa and widely cultivated in the tropical and subtropical zones of the world, cowpea (*Vigna unguiculata* (L.) Walp.), also known as black‐eyed pea, is one of the most valuable grain legumes for high‐quality dietary protein, carbohydrates, lipids, minerals and vitamins for people in developing countries of Africa and Asia (Abdu Sani *et al*., 2015; Phillips *et al*., 2003; Singh, 2014). It is estimated that over 200 million people consume cowpea daily in Africa (Phillips *et al*., 2003; Singh, 2014). Despite its high tolerance to heat, dry conditions and soil acidity, cowpea is highly susceptible to insect pests and pathogen infestations, resulting in lower productivity (Abdu Sani *et al*., 2015; Boukar *et al*., 2016; Obembe, 2008; Singh, 2014; Solleti *et al*., 2008a). Due to limited genetic variability of cowpea and strong cross‐incompatibility between wild *Vigna* species and cultivated cowpea, little progress has been made in genetic improvement through conventional breeding to achieve desirable agronomic traits (Abdu Sani *et al*., 2015; Fang *et al*., 2007; Gomathinayagam *et al*., 1998; Latunde‐Dada, 1990; Wamalwa *et al*., 2016). Hence, plant biotechnology provides an alternative approach to overcome those constraints for improving the agronomic performance and developing improved cowpea cultivars with higher grain quality and yield (Carlos Popelka *et al*., 2004; Zaidi *et al*., 2005). The development of insect‐resistant cowpea, unsuccessful through conventional breeding, was successfully achieved by introducing *Bt* genes through genetic transformation and is a good example of plant biotechnology application in an orphan crop (Bakshi *et al*., 2011; Bett *et al*., 2017; Zaidi *et al*., 2005). Recently, significant progress has been made establishing genomic and gene expression data resources for two cowpea varieties, IT86D‐1010 (Spriggs *et al*., 2018) and IT97K‐499‐35 (Lonardi *et al*., 2019; Munoz‐Amatriain *et al*., 2017; Yao *et al*., 2016). However, the absence of an efficient genetic transformation and editing system (Popelka *et al*., 2006; Somers *et al*., 2003) has impeded the full utilization of these resources for cowpea functional genomic studies to elucidate the mechanisms of heat and drought stress tolerance and to improve the agronomic traits, such as insect and pathogen resistances and increased productivity.

Legumes, especially cowpea, are known to be recalcitrant for genetic manipulation (Manman *et al*., 2013; Popelka *et al*., 2006; Solleti *et al*., 2008b; Somers *et al*., 2003). This is mainly due to the inadequate *Agrobacterium*‐mediated transfer DNA (T‐DNA) delivery to the targeted tissue, the inefficient transgenic selection methods for viable transgenic plant recovery and the absence of an amenable *in vitro* shoot regeneration system. Although notable improvements have been made in recent years for *Agrobacterium*‐mediated cowpea transformation using two types of explants, the cotyledonary node (cot‐node) explants excised from germinated seedlings (Bakshi *et al*., 2011) and the cotyledon with attached embryonic axis (EA) from imbibed mature seeds (Bett *et al*., 2019), the published transformation frequencies (Bett *et al*., 2019) are still lower than 3.9% (Bett *et al*., 2019; Chaudhury *et al*., 2007; Manman *et al*., 2013; Mellor *et al*., 2012) and the tissue culture process generally requires excessive explant manipulation to remove the cotyledon, primary shoots and any regrown radicle and usually takes at least 5 to 8 months for generating fully developed plantlets after *Agrobacterium* infection (Chaudhury *et al*., 2007; Popelka *et al*., 2006). Besides different explant types, several selection systems have been reported for cowpea transformation (Manman *et al*., 2013), such as *NPTII*/kanamycin (Bett *et al*., 2019; Chaudhury *et al*., 2007), *NPTII*/G418 (Solleti *et al*., 2008b), *PMI*/mannose (Bakshi *et al*., 2012), *HPT*/hygromycin (Kumar *et al*., 1996), *BAR*/glufosinate (Popelka *et al*., 2006) and *ahas*/imazapyr (Citadin *et al*., 2013; Ivo *et al*., 2008). It was reported that incomplete selection and tissue necrosis were associated with those selection systems (Manman *et al*., 2013) and resulted in lower transgenic plant recovery (Bakshi *et al*., 2011; Chaudhury *et al*., 2007; Solleti *et al*., 2008b) and a higher percentage of chimera formation in cowpea (Das Bhowmik *et al*., 2019).

To increase the regeneration rate, enhance transgenic plant recovery and eliminate chimera formation under selection in this study, we first evaluated shoot regeneration using detached EA explants isolated from imbibed mature (dry) seeds and identified that only cot‐node cells of the detached EA explants undergo the rapid cell division and dedifferentiation required to acquire organogenic competence for shoot regeneration. Based on this observation, we developed a rapid, robust and highly efficient *Agrobacterium*‐mediated EA‐based transformation using *CTP‐spcN* as selectable marker and generated transgenic cowpea events without non‐transgenic escapes and chimera formation. Finally, we applied this transformation technology to nine cowpea genotypes and achieved transformation frequencies in the range of 4% to 37% and demonstrated its potential to support efficient genome editing by creating inheritable knockouts in IT86D‐1010 using CRISPR/Cas gene editing technology. The overall tissue culture process after *Agrobacterium* infection to generate fully developed plantlets was reduced to less than 3 months.

## RESULTS AND DISCUSSION

### *De novo* shoot organogenesis using EA as explants

**Figure 1:**
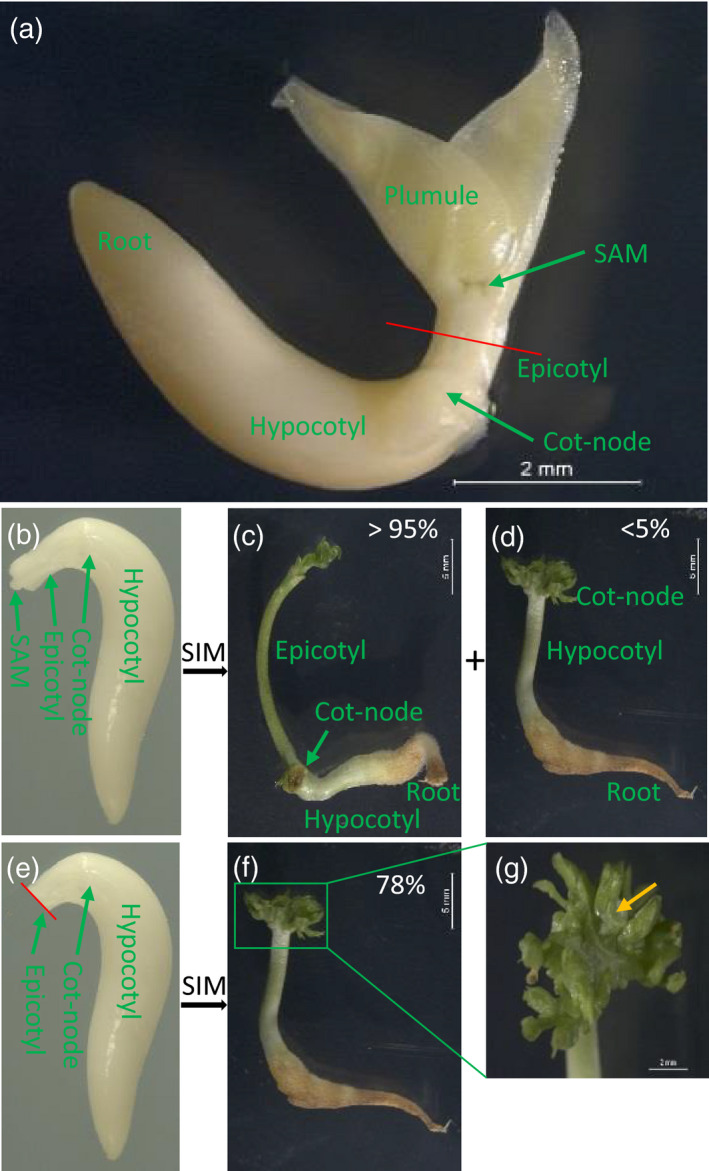
The regeneration origin of cowpea EA explants *via* organogenesis. (a) The structure of cowpea IT86D‐1010 EA extracted from imbibed mature seeds. (b–d) The single (c) and multiple (d) shoot development from non‐decapitated EA explants with the plumule excised (b). (e,f) The multiple shoot regeneration (f) from decapitated EA explants (e). (g) Enlarged view of multiple shoot regeneration and the cutting site of the epicotyl indicated by the yellow arrow. The percentages in (c), (d) and (f) represent the rates of single and multiple shoot development determined from 100 non‐decapitated and 100 decapitated EA explants after 10 days on SIM. The red line through the middle of the epicotyl represents the decapitation process of EA explants to remove the SAM.

A rapid, efficient and reproducible regeneration system is a prerequisite for establishment of an efficient cowpea genetic transformation system. Although several studies of *in vitro* regeneration of cowpea based on organogenesis have been reported (Aasim *et al*., 2010; Abdu Sani *et al*., 2015; Mamadou *et al*., 2008; Manman *et al*., 2013; Odutayo *et al*., 2005; Raveendar *et al*., 2009; Sani *et al*., 2018; Tie *et al*., 2013; Yusuf *et al*., 2008), an efficient cowpea regeneration system that enables highly efficient transformation is still lacking (Manman *et al*., 2013). Soybean (*Glycine max*) transformation based on the pre‐existing meristems of EA explants has been well established and provides a reliable and highly efficient means for introducing transgenes (Aragão *et al*., 2000; Liu *et al*., 2004; Turlapati *et al*., 2008). To test the regeneration efficiency of EA explants in cowpea, EA explants were isolated from imbibed mature seeds of cowpea variety IT86D‐1010 by excising the cotyledons at the cot‐nodes (Figure 1(a)). Those EA explants with the plumule excised (Figure 1(b)) were then cultured directly onto shoot induction medium (SIM) (Table S5) without selection in a vertical upright position with roots embedded in the media to induce shoot development. In most of the cases (>95%), a single primary shoot was developed per EA when the shoot apical meristem (SAM) of the EA explants was kept intact (non‐decapitated EA explants) during regeneration (Figure 1(c)). However, multiple shoot development was observed occasionally for a small number of explants (<5%) (Figure 1(d)). Compared to the morphology of EA explants with single primary shoot development (Figure 1(c)), the EA explants with multiple shoots regenerated exclusively around cot‐nodes (Figure 1(d)), were much shorter and lacked both epicotyls and SAMs. The lack of epicotyls and SAMs could be due to the accidental damage of those tissues during EA isolation and plumule excision. This finding indicated that the *de novo* organogenesis of shoots around the cot‐nodes could be inhibited by apical dominance. Indeed, removal of the SAM purposely by cutting through the middle of each epicotyl (decapitated EA explants without SAM and plumule) (Figure 1(a,e)) induced 78% of explants to initiate multiple shoot regeneration in IT86D‐1010 (Figure 1(f,g) and Table S9).

To test if the regeneration principle described above was applicable to other cowpea germplasm accessions and even common bean (*Phaseolus vulgaris* L.), we further tested tissue culture and *in vitro* regeneration procedures for eight additional cowpea accessions from the U.S. National Plant Germplasm System (NPGS), two non‐conventional cowpea germplasm lines (TPC‐001 and MRS‐001) and two common bean varieties, *black* bean (CBB‐001) and *pinto* bean (CBP‐001), collected from tropical and subtropical regions of Mexico (Figure S3). Consistent with observations in IT86D‐1010, shoot regeneration also developed exclusively from cot‐node regions for all 10 cowpea germplasm lines and two bean germplasm lines tested (Figure S4). In most cases, the number of EA explants showing multiple shoot regeneration exceeded those showing a single regenerated shoot, suggesting that multiple individuals can be recovered from a single EA explant (Table S9 and Figure S4). The overall regeneration efficiency ranged from 55% to 81% for eight additional cowpea accessions from the NPGS, ranged from 36% to 38% for Mexican cowpeas and was 30% for common beans (Table S9). These results demonstrated that tissue culture and regeneration procedures can be applied to a wide collection of cowpea germplasm and extended to other legumes such as common bean.

### Regeneration optimization under *Agrobacterium*‐mediated transformation

Generally, the EA‐based dicot transformation procedure consists of the following main steps: explant preparation, *Agrobacterium* infection, co‐cultivation, shoot regeneration with selection and root induction (Figure S2 and the Experimental Procedures section). As described above, although EA explants *per se* have been described for soybean transformation, the cowpea EA‐based regeneration system has a key difference. While the soybean EA transformation system relies on pre‐existing apical meristematic tissue for regeneration and transformation (Aragão *et al*., 2000; Liu *et al*., 2004; Turlapati *et al*., 2008), the cowpea regeneration system shows that removal of the SAM stimulated multiple shoot organogenesis from the cot‐node. This key difference raises the question of how well the decapitated EA explants will be able to survive and regenerate throughout the *Agrobacterium*‐mediated transformation procedure.

**Figure 2:**
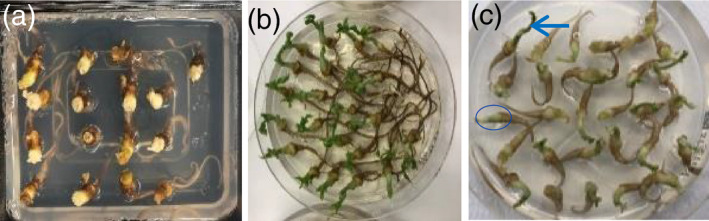
Explant sensitivity to infection and co‐cultivation treatments. (a,b) The survival and regeneration capability of decapitated (a) and non‐decapitated (b) EA explants after 10‐day shoot induction without selection following infection and co‐cultivation steps without *Agrobacterium* inoculation. (c) The susceptibility of non‐decapitated EA explants to *Agrobacterium* inoculation following co‐cultivation. The arrow and the circle indicate one of the surviving explants with elongated epicotyl and one of the dying explants without elongated epicotyl, respectively. Image was taken after 4 days on SIM.

To evaluate how the decapitation of EA explants affects the survival and regeneration capability, we conducted sonication, *Agrobacterium* infection and co‐cultivation treatments (Figure S2 and the Experimental Procedures section) either with or without *Agrobacterium*, followed by regeneration on SIM. As shown in Figure 2(a), the decapitated EA explants were extremely sensitive to the treatments and none of the EA explants survived on SIM without selection after mimicking all the treatment steps without *Agrobacterium*. On the contrary, all the non‐decapitated EA explants survived and formed elongated epicotyls with a single primary shoot. The further decapitation of those primary shoots by cutting through the middle of the elongated epicotyls after 4 days of regeneration stimulated multiple shoot organogenesis around the cot‐nodes (Figure 2(b)). Collectively, those observations suggest that although the SAM negatively regulates multiple shoot organogenesis from cot‐node tissues because of the apical dominance effect, the SAM is essential for EA explant survival through the transformation treatments before regeneration and the SAM should not be removed until the explants are fully recovered after 4 days of regeneration.

It has been reported that *Agrobacterium*‐mediated infection leads to cell damage and tissue necrosis (Norkunas *et al*., 2018). To determine the survival rate of EA explants after infection and co‐cultivation with *Agrobacterium*, non‐decapitated EA explants without plumules were subjected to the transformation procedure (Figure S2 and the Experimental Procedures section) using LBA4404 Thy‐ carrying the pPHP86170/pPHP71539 vector system as described below. As shown in Figure 2(c), about 70±10% EA explants (based on the average of three replicates and 75 EA explants) survived and formed elongated epicotyls after 4‐day regeneration on SIM with selection (Table S5). Compared to the 100% survival rate without *Agrobacterium* inoculation (Figure 2(b)), the 30% mortality rate was most likely due to the sensitivity of EA explants to the *Agrobacterium*.

It is known that plumules interfere with shoot regeneration and need to be removed from soybean EA explants. The same is true for cowpea EA explants. No transgenic shoots can be regenerated without removing the plumule. However, removing plumules from isolated EAs, one by one very carefully without damaging the SAM, is the most time‐consuming and labor‐intensive step of this process. This is because EA isolation with an intact SAM is the critical step for maximizing explant survival during tissue culture inoculation as described above. To avoid SAM damage during EA explant preparation and for the purpose of developing a best practice, we simplified the EA isolation and subsequent transformation procedure as follows. Instead of removing the plumule at the beginning of EA isolation, as usually performed for soybean, both the SAM and the plumule are removed simultaneously on the fourth day on SIM by cutting through the middle of the epicotyl with a pair of surgical scissors (Figure S2 and the Experimental Procedures section). This improved procedure was implemented for all the subsequent transformation optimization experiments throughout this study.

### *Agrobacterium*‐mediated gene delivery using a ternary vector system

A dramatic increase of T‐DNA delivery efficiency was reported in cowpea by constitutive expression of additional *vir* genes in a resident pSB1 vector in *Agrobacterium* strain LBA4404 (Solleti *et al*., 2008b). Recently, we demonstrated that a newly designed ternary vector containing the T‐DNA binary vector and the optimized pVIR accessory (pPHP71539) plasmid with additional *vir* genes enhanced gene delivery and ultimately the transformation efficiency for both corn (*Zea maize*) and sorghum (*Sorghum bicolor*) (Anand *et al*., 2018; Che *et al*., 2018). This encouraged us to assess the gene delivery efficiency of the ternary vector for cowpea transformation.

**Figure 3:**
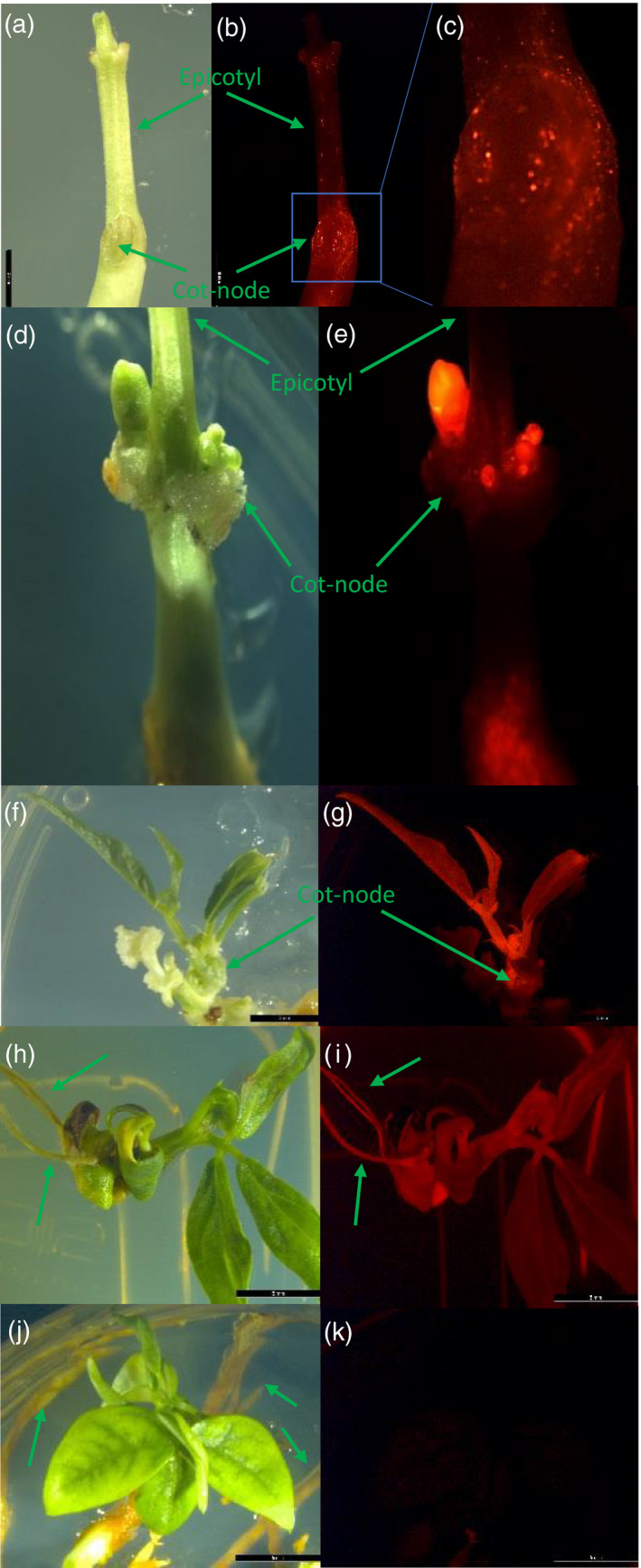
Different stages of transgenic cowpea IT86D‐1010 development using the CTP‐spcN/SPEC selection system. (a,b) T‐DNA delivery, as determined by transient assay after 3 days on SIM. (c) Enlarged view of the size, the intensity and the amount of fluorescent foci around the cot‐node region. (d,e) Transgenic shoot budding after 2 weeks of regeneration on SIM with SPEC selection. (f,g) Fully developed transgenic shoot after 5 weeks of regeneration with SPEC selection. (h,i) Root development of regenerated transgenic shoot after 3 weeks of root induction on RIM. (j,k) Autofluorescence evaluation of regenerated wild‐type cowpea IT86D‐1010. The arrows indicate the regenerated roots. (a,d,f,h,j) Bright field images. (b,c,e,g,i,k) Fluorescence images under RFP filter.

To evaluate T‐DNA delivery using the ternary vector system in cowpea, we transformed binary vector pPHP86170 (Figure S1(a)) containing the *proDMMV:TagRFP* as the visual marker and *proGM‐UBQ:CTP‐spcN* (GenBank Accession No. AAD50455) (Anada *et al*., 2017) as the selectable marker into the *Agrobacterium* strain LBA4404 Thy‐ harboring the pVIR accessory plasmid pPHP71539. Transient gene delivery was assessed by visually evaluating the number of fluorescent foci on the surface of cowpea EA explants after 3 days of shoot induction on SIM containing spectinomycin (SPEC) for selection. As shown in Figure 3(b), strong gene delivery based on the number of infected cells was visualized across the entire explant for those surviving EA explants with elongated epicotyls (Figure 3(a,b)), especially around the cot‐node tissue (Figure 3(b,c)), demonstrating highly efficient gene delivery in cowpea EA explants. Although gene delivery was efficient across the entire EA explant, only those fluorescent foci within the cot‐node tissue showed subsequent development and substantially enhanced fluorescence intensity during regeneration (Figure 3(b,c)). This observation supported the hypothesis that only those cells within the cot‐node region, but not any other tissues of the EA explant, actively undergo rapid cell division and dedifferentiation to acquire organogenic competence for shoot regeneration.

### *In vitro* regeneration and transgenic selection of cowpea

Several selection systems have been reported for cowpea transformation with different explant types (Manman *et al*., 2013), such as *NPTII*/kanamycin (Bett *et al*., 2019; Chaudhury *et al*., 2007), *NPTII*/G418 (Solleti *et al*., 2008b), *PMI*/mannose (Bakshi *et al*., 2012), *HPT*/hygromycin (Kumar *et al*., 1996), *BAR*/glufosinate (Popelka *et al*., 2006) and *ahas*/imazapyr (Citadin *et al*., 2013; Ivo *et al*., 2008). It was reported that incomplete selection and tissue necrosis were associated with those selection systems (Manman *et al*., 2013) and resulted in lower transgenic plant recovery (Bakshi *et al*., 2011; Chaudhury *et al*., 2007; Solleti *et al*., 2008b) and a higher percentage of chimeric tissue formation in cowpea (Das Bhowmik *et al*., 2019). To identify more efficient selection agents for the *de novo* organogenesis described above, we tested and compared the selection efficiency of *CTP‐NPTII*/kanamycin, *CTP‐NPTII*/G418 and *CTP‐spcN*/SPEC (Anada *et al*., 2017) after *Agrobacterium*‐mediated transformation using *Agrobacterium* strain LBA4404 Thy‐. Ideally, the optimal concentration for effective selection is determined by testing different concentrations of selective reagents in SIM that completely inhibits the *in vitro* regeneration from the wild‐type EA explants, but imposes minimal or no impact on transgenic shoot recovery and development. Since a *CTP‐spcN*/SPEC selection system for EA‐based soybean transformation has been well established and implemented for soybean transformation at a SPEC concentration of 25 mg L^−1^ in Corteva, we decided to test three SPEC concentrations (15, 25 and 35 mg L^−1^) for cowpea transformation and observed similar efficacy in terms of high transgenic shoot recovery with no transgenic escapes. Therefore, we chose a SPEC concentration of 25 mg L^−1^ throughout the study. As described above, strong gene delivery was observed around the cot‐node target tissue on the third day of regeneration as shown in Figure 3(b,c). Those fluorescent foci grew quickly, and single or multiple shoot buds emerged exclusively around the cot‐nodes within 2 weeks following removal of the SAM on the fourth day of regeneration (Figure 3(d,e)). Transgenic shoots were fully developed from the buds within another 3 weeks in which all the shoots displayed strong fluorescence evenly across the entire regenerated shoot (Figure 3(f,g)) compared to the regenerated shoots from wild‐type cowpea explants that showed no fluorescence at all (Figure 3(j,k)). The elongated shoots were excised from the EA explants and transferred to root induction medium (RIM) (Table S6) for root development. Because of the stringent selection during shoot organogenesis, selection was not required for rooting. Approximately 95% (Table 1) of the elongated shoots fully rooted in the RIM within 2–3 weeks and all the regenerated shoots and roots displayed strong fluorescence (Figure 3(h,i)). The total time from inoculation of the EA explants to transplantation of a fully developed transgenic plantlet in the greenhouse took approximately 2–3 months. As shown in Table 1, the frequency of shoot formation was about 21% for IT86D‐1010, of which about 23% of the events were single copy quality events (see the Experimental Procedures section for the definition and determination of ‘quality events’).

**Table 1:** Transformation efficiency and T0 event quality for IT86D‐1010 using pPHP86710

# of EAs	# of transgenic shoots	# of chimeric shoots*	# of transgenic shoots with root developed	Rooting efficiency (%)	Transformation efficiency (%)	Quality events (%)
30	7	0	6	86	20	23
30	7	0	7	100	23	
20	4	0	4	100	20	
				95±8**	21±2**	

* Chimeric shoots were identified by the presence of distinct sectors of cells exhibiting fluorescence, which were sharply demarcated from non‐fluorescent tissue.

** Data are presented as the average rooting and transformation efficiencies ± SD of three biological replicates.

Chimeric tissue formation (a single plant tissue containing a mixture of transformed and non‐transformed sections) during tissue culture transformation is a prevalent issue in legumes, including cowpea (Das Bhowmik *et al*., 2019). Chimeric plants affect the segregation of the transgene to the subsequent generation and reduce the efficiency of recovering stable transgenic lines (Das Bhowmik *et al*., 2019). Reporter genes, such as *uidA* (encoding GUS) (Das Bhowmik *et al*., 2019), *GFP* (Dutt *et al*., 2007) and *Ds‐RED* (Xu *et al*., 2020), are often used to determine the uniformity of gene expression on the regenerated shoots for chimera formation. In this study, the formation of chimerism was evaluated for all the transgenic events generated using binary vector pPHP86170 (Figure S1(a)) containing the *proDMMV:TagRFP* as the visual marker and pPHP92782 (Figure S1(c)) containing the *proGM‐EF1A2:Ds‐RED* as the visual marker. In addition to the 18 transgenic shoots reported in Table 1, an additional 234 regenerated shoots generated using pPHP86170 (Figure S1(a)) and 59 regenerated shoots generated using pPHP92782 (Figure S1(c)) were screened and showed no signs of chimerism. Therefore, the utilization of the *CTP*‐*spcN*/SPEC system provided more efficient and stringent transgenic selection and generated transgenic cowpea events with neither non‐transgenic escapes nor chimera formation. Conversely, as shown below in Table 3, a chimerism percentage in the range of 17% to 33% (Table 3) was observed when a hypervirulent *Agrobacterium* strain, AGL1 (Lazo *et al*., 1991), and an alternate SPEC selection system, *CTP‐aadA*/SPEC (Martinell *et al*., 2017), were utilized.

In contrast to SPEC as the selection reagent, cowpea IT86D‐1010 EA explants showed a high degree of resistance to kanamycin and no selection pressure could be built up by culturing wild‐type EA explants on SIM containing kanamycin at concentrations as high as 600 mg L^−1^. Although the optimal concentration of G418 at 20 mg L^−1^ for selection of transformed shoots was established by culturing non‐transformed EA explants on SIM containing different concentrations of G418 (10–40 mg L^−1^), the G418 selection was not as stringent as for SPEC. As indicated by the uneven and partial fluorescence of the regenerated shoot in Figure S5, all five events generated from 110 EA explants were chimeric events using the binary vector pPHP94518 (Figure S1(b)) containing *proGM‐EF1A2:Ds‐RED* as a visual marker and *proGM‐UBQ:CTP‐NPTII* as the selectable marker. Because of the low transformation efficiency and high chimera formation rate under G418 selection, we stopped conducting further optimization and concluded that *CTP‐NPTII*/kanamycin and *CTP‐NPTII*/G418 were not efficient selection systems for cowpea EA‐based transformation.

### Transgene inheritance in the progeny

To evaluate the inheritance of T‐DNA integration events, we selected nine independent single copy quality T0 transgenic events (lines) in the IT86D‐1010 background transformed with construct pPHP92782 (Figure S1(c)) containing *proGM‐EF1A2:Ds‐RED* as a visual marker gene and *proGM‐UBQ:CTP‐spcN* as the selectable marker gene. Those T0 plants with chimera formation based on *Ds‐RED* expression were self‐pollinated in the greenhouse and the resultant T1 seeds displayed various levels of red color (Figure S6), because the overall intensity of the seed color is largely represented by the color of cotyledons passing through the semi‐translucent seed coat, as described by Nishizawa *et al*. in soybean carrying the *Ds‐RED* transgene (Nishizawa *et al*., 2006). Therefore, the variation of seed color intensity in T1 cowpea seeds could represent the level of transgene expression in the cotyledon and indicate transmission and segregation of the transgene in the progeny. To further determine the segregation ratio, T1 seeds from each of the nine independent events were randomly chosen regardless of seed color and advanced to the T1 generation. The zygosity (homozygous, hemizygous and null) of segregated T1 plants was characterized by determining the copy number of the integrated T‐DNA based on the assays described in the Experimental Procedures section. As shown in Table 2, the segregation pattern of eight out of nine transgenic events showed a typical 1:2:1 Mendelian ratio in the T1 generation based on chi‐square test analysis, except event 125739949, which had a *P*‐value of 0.04, which was barely lower than the threshold at 0.05. Nonetheless, 28 homozygous plants were still identified from 98 segregated T1 plants in this event. These results demonstrate that all the transgenic events analyzed possessed stably integrated T‐DNA without obvious chimera formation and the T‐DNA was successfully passed on to the next generation.

**Table 2:** Segregation analysis of self‐fertilized IT86D‐1010 transgenic cowpea plants in the T1 generation

Event ID	Total # of plants analyzed	# of two copy plants^1^	# of single copy plants^1^	# of null plants^1^	Expected segregation ratio	Chi‐square test	*P*‐value^2^	H_0_ hypothesis^3^
125739938	100	17	54	29	1:2:1	3.52	0.17	Accepted
125739949	98	28	37	33	1:2:1	6.39	0.04	Rejected
125739950	190	41	106	43	1:2:1	2.59	0.27	Accepted
125739956	94	19	49	26	1:2:1	1.21	0.55	Accepted
125739954	92	25	44	23	1:2:1	0.88	0.88	Accepted
135577762	84	19	46	19	1:2:1	0.76	0.68	Accepted
135577763	82	21	41	20	1:2:1	0.024	0.99	Accepted
135577764	88	28	39	21	1:2:1	2.25	0.32	Accepted
135577766	73	15	45	13	1:2:1	4.06	0.13	Accepted

^1^ Copy numbers were determined by *PSB1*, *PSA2*, *Ds‐RED* and *spcN‐SO* qPCR assays (Experimental Procedures).

^2^ The observation ratio is considered to fit the expected segregation ratio of 1:2:1 if *P* > 0.05.

^3^ H_0_ hypothesis: The segregated T1 population fits the 1:2:1 genotype ratio.

### Transformability evaluation of different cowpea genotypes

Genotype dependence is a major limitation of regeneration and *Agrobacterium*‐mediated transformation for both monocots and dicots (Abdu Sani *et al*., 2015; Che *et al*., 2018; Jia *et al*., 2015; Manman *et al*., 2013). To evaluate the robustness of the protocol and broaden the application of this cowpea transformation technology for different cowpea genotypes, we performed a quick transformability assay to evaluate the formation of fluorescent transgenic shoots after 2 weeks of culture on SIM with selection. This quick transformability assay was conducted using a hypervirulent *Agrobacterium* strain AGL1 carrying the RC2717 plasmid with *CTP‐aadA* as the selectable marker (Figure S1(d)). As shown in Table 3, the transgenic shoot regeneration frequency (defined as transformability) of IT86D‐1010, as determined by the quick transformability assay, was in the range of 11% to 26% (average, 19±7.5%) (Table 3). This was comparable to the 21±2% transformation efficiency described earlier using LBA4404 Thy‐ carrying the pVIR accessory plasmid for transformation and *CTP‐spcN*/SPEC for selection (Table 1), indicating the reliability of this quick assay for predicting transformation efficiency of different genotypes. The application of this quick transformability assay to eight more cowpea genotypes showed transformability rates ranging from 4% to 37% (Table 3). Similar to the observation of transgenic shoot development for IT86D‐1010, all eight cowpea genotypes also formed transgenic shoots exclusively and rapidly at the cot‐node region and, in most of the cases, no more than two transgenic shoots per explants were developed (Figure S7). These results demonstrate that the transformation protocol developed for IT86D‐1010 described herein is transferable to other genotypes even with an alternative *Agrobacterium*‐mediated transformation system. However, a high percentage of chimerism (in the range of 17% to 33%) (Table 3) was observed using this quick transformability assay, most likely attributable to the hypervirulent AGL1 *Agrobacterium* strain and/or the *CTP‐aadA*/SPEC selection system.

**Table 3:** Transformability evaluation of nine cowpea accessions

Germplasm	# of EAs	# of EAs with fluorescent shoots at 2 weeks	Transformability^1^ (%)	# of EAs with fluorescent shoots at 4 weeks	# of chimeric shoots^2^	Chimerism rate^3^ (%)
Experiment 1						
IT86D‐1010	123	14	11	24	7	29
PI 527675	166	52	31	34	9	26
PI 580227	178	10	6	12	4	33
TVu 9693	160	35	22	43	9	21
Experiment 2						
IT86D‐1010	117	22	19	43	11	26
PI 583259	119	31	26	41	7	17
TVu 79	157	7	4	22	6	27
TVu 8670	125	11	9	13	3	23
Experiment 3						
IT86D‐1010	102	27	26	NA	NA	NA
PI 582835	90	33	37	NA	NA	NA
TVu 3562	177	32	18	NA	NA	NA

NA, not available.

^1^ Transformability is defined as the number of EAs with fluorescent shoots divided by the total number of EA explants.

^2^ Chimeric shoots were identified by the presence of distinct sectors of cells exhibiting fluorescence, which were sharply demarcated from non‐fluorescent tissue.

^3^ Chimerism rate is defined as the number of chimeric shoots divided by the total number of regenerated shoots with fluorescence at 4 weeks after transformation.

In general, a better shoot organogenesis response tends to produce a higher transgenic shoot regeneration frequency, but this is not always the case. As shown in Tables 3 and S9, although all five genotypes, IT86D‐1010, PI 527675, PI 580227, PI 582835 and TVu 79, showed very good and comparable shoot organogenesis rates (in the range of 78% to 81%), only IT86D‐1010, PI 527675 and PI 582835 demonstrated significant high transformability efficiencies (19±7.5%, 31% and 37%, respectively), in contrast to PI 580227 and TVu 79 (5.6% and 4.5%, respectively). In contrast, all three genotypes, TVu 3562, TVu 9693 and PI 583259, had relatively low shoot organogenesis rates (68%, 61% and 56%, respectively) (Table S9), but their transformability efficiency was relatively high (18%, 22% and 26%, respectively) (Table 3). Therefore, the transformability of those germplasm lines is determined not only by the shoot organogenesis capability, but also by the combination of the susceptibility to *Agrobacterium*‐mediated T‐DNA delivery and sensitivity to *Agrobacterium* infection and sonication‐related damage.

### Targeted cowpea genome editing with CRISPR/Cas9

**Figure 4:**
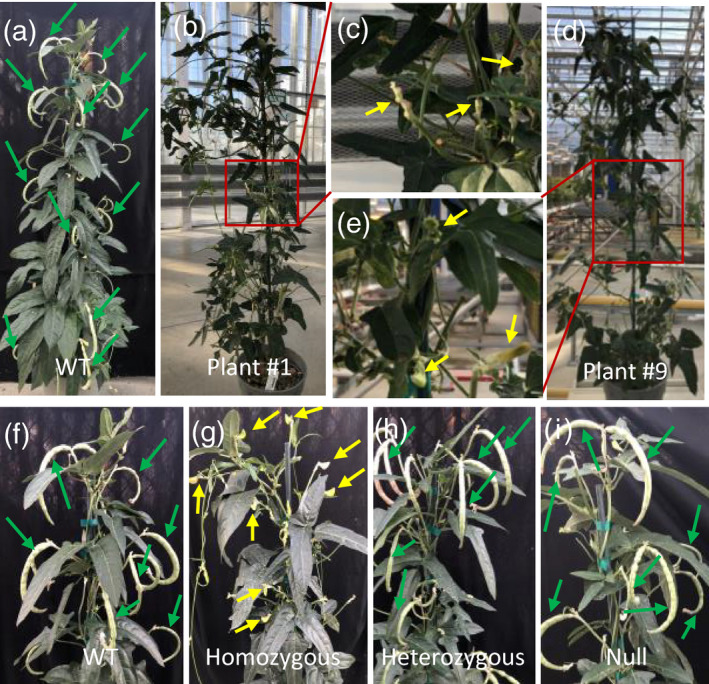
Fertility phenotypes. (a,f) The fertile phenotype of wild‐type cowpea IT86D‐1010 with mature pod development. (b–e) The infertile phenotype of T0 biallelic *Vu‐SPO11* mutants, plant #1 (b) and plant #9 (d). (c,e) Enlarged view of a portion of biallelic *Vu‐SPO11* mutants, plant #1 (b) and plant #9 (c). (g–i) Fertility phenotypes of the T1 segregants, homozygous (g), heterozygous (h) and null mutant (i), of edited T0 plant #26. Seed pods are indicated by green arrows. Floral sectors with pod abortion are indicated by yellow arrows.

The lack of mutation resources and efficient means for gene inactivation has dramatically hampered the genetic improvement of cowpea for breeding. Targeted genome editing using the CRISPR/Cas system has proven to be a powerful tool for crop engineering and has been successfully applied to maize, sorghum, soybean, rice (*Oryza sativa*) and numerous other plant species to generate stable genome‐edited lines with targeted modifications (e.g., insertions, deletions and replacements). Recently, CRISPR/Cas‐mediated genome editing in cowpea was demonstrated in non‐inheritable mutated hairy roots (Ji *et al*., 2019) and stable, heritably edited, mutated plants (Juranic *et al*., 2020). To establish the CRISPR/Cas‐mediated genome editing system in cowpea using the transformation protocol described herein and to test the feasibility for highly efficient editing, we designed a new cowpea gene editing construct, pPHP96249, carrying *spCas9* driven by a soybean elongation factor *EF1A2* promoter (Li *et al*., 2015) and the Vu‐SPO11‐CR2 single guide RNA (sgRNA) driven by the cowpea U6 promoter (*Vu‐U6.1*) (Juranic *et al*., 2020), to knock out the cowpea meiosis gene *Vu‐SPO11‐1* (Juranic *et al*., 2020) (Figures S1(e) and S8 and the Experimental Procedures section). A total of 35 independent T0 transgenic plants were obtained from 250 EAs in the IT86D‐1010 background. All the T0 plants were analyzed by deep sequencing to identify mutations at the target site (Table S12 and the Experimental Procedures section). As shown in Table 4 and Table S12, a genome editing efficiency of 68.6% was observed, in which 25.7% of the edited plants showed an edited read percentage higher than 30% of at least one allele. A higher allele read percentage generally indicates higher allele recovery potential in the next generation. This large population of T0 plants with high editing efficiency provided us with the opportunity to investigate the types of edit mutations in cowpea for the first time. As shown in Table 4 and Table S12, 70.8% of edited plants carried a 1‐bp G deletion and 33.3% of the edited plants carried a multiple‐bp deletion. The only type of insertion detected by deep sequencing was a 1‐bp G insertion, at a frequency of 37.5%. Thus, deletions are the predominant type of edited mutation, especially the single base pair G deletion (70.8%), which results in a frameshift or knockout of the gene. Among the edited plants, 41.6% carried more than one edited allele forming chimeric mutations (Table 4). Remarkably, two biallelic knockouts (plants #1 and 9) were identified from 35 T0 transgenic plants by deep sequencing (Table 4 and Table S12). Plant #1 contained a 1‐bp G deletion on both alleles (94% of allele reads) and plant #9 had two types of mutated alleles, a 1‐bp G insertion (42% of allele reads) and a 22‐bp deletion (52% of allele reads), all leading to a null mutation of *Vu‐SPO11* (Table S12). Those two biallelic knockouts allowed us to characterize mutant phenotypes in the T0 generation. As shown in Figure 4(b–e), both T0 biallelic mutants showed complete infertility with no seed pods developed on the plants. On the contrary, all the other T0 heterogeneous mutations were totally fertile and produced pods and seeds. To demonstrate that the edited allele can be stably transmitted and to further confirm the fertility phenotype of *Vu‐SPO11* mutation in the subsequent generation, we characterized the target gene mutation in the progenies from three selected heterogeneous mutation T0 events (#26, #28 and #29) (Table S12 and Table 5). As shown in Table 5, T1 heterozygous mutations and null mutations were recovered in the progenies of all three selected T0 events, and T1 homozygous mutations were identified in the progenies of both event #26 and event #28. These results demonstrate that the edited alleles are heritable and can be segregated to form heterozygous, homozygous and null genotypes in the next generation. Consistent with the observation of the two T0 biallelic knockouts (Figure 4(b–e)), all T1 homozygous identified in Table 5 were completely infertile with aborted seed pod development through plant maturity (Figure 4(g)). Contrarily, all the segregated null mutants and heterozygous plants were fertile with normal seed pod development and not distinguishable from the wild‐type (Figure 4(a,f,h,i)). These observations further supported the functionality of *Vu‐SPO11* in meiosis, as characterized by Juranic *et al*. (Juranic *et al*., 2020). Overall, these results demonstrated that we have established a highly efficient CRISPR/Cas9‐mediated editing system for selectively altering genome DNA sequences in cowpea.

**Table 4:** CRISPR/Cas9‐mediated gene editing efficiency and types for *Vu‐SPO11* in IT86D‐1010

Total # of T0 plants	# of T0 edited plants^1^	# of T0 edited plants with allele read percentage > 30%^2^	# of T0 edited plants with biallelic knockouts^3^	# of T0 edited plants with chimeric edits (≥2 edited alleles)	# of T0 edited plants with multiple‐bp deletion	# of edited T0 plants with 1‐bp G deletion	# edited T0 plants with 1‐bp G insertion
35	24 (68.6%)^4^	9 (25.7%)^4^	2 (5.7%)^4^	10 (41.6%)^5^	8 (33.3%)^5^	17 (70.8%)^5^	9 (37.5%)^5^

^1^ Edits are defined as mutagenesis at target site.

^2^ See Table S12 for the allele read percentage in each plant.

^3^ Biallelic knockout is defined as targeted mutagenesis that results in a frameshift mutation for both alleles.

^4^ The number in parentheses represents the frequency of the mutation type in the total T0 plants.

^5^ The number in parentheses represents the frequency of the mutation type in the total edited plants detected.

**Table 5:** The heritability of mutations in T1 plants of IT86D‐1010

Event ID	Type of edits in T0 progenitors	# of T1 plants analyzed	# and genotypes of T1 plants harboring homozygous mutations from T0 plants^1^	# and genotypes of T1 plants harboring heterozygous mutations from T0 plants^1^	# and genotypes of T1 plants harboring chimeric mutations from T0 plants^1^	# of null plants segregated from T0 plants^1^
26	+G (33%); WT (57%)	35	11; +G/+G	14; +G/WT	Not relevant	10; WT/WT
28	−G (26%); +G (8%); −GC (8%); −CGTG (5%); WT (19%)	25	1; −G/−G	4; −G/WT	0	10; WT/WT
			1; +G/+G	9; +G/WT		
			0; −GC/−GC	0; −GC/WT		
			0; −CGTG/−CGTG	0; −CGTG/WT		
29	−G (25%); +G (6%); WT (25%)	18	0; −G/−G	2; −G/WT	1; −G (11%); +G (8%); WT (47%)	11; WT/WT
			0; +G/+G	4; +G/WT		

The number in parentheses represents the frequency of the mutation type in the total edited plants detected. If no percentage is shown, the one genotype is around 100% and the two genotypes are around 50%:50%.

^1^ For each T1 plant, only the genotypes of their T0 progenitors are characterized.

In summary, we have developed a rapid, robust, flexible and highly efficient cowpea transformation and CRISPR/Cas9‐mediated genome editing system using EA as explants. The principles established in this study have the potential to increase the transformation efficiencies of other legume species and, potentially, other dicot crops. With recent progress establishing cowpea genetic and genomic resources (Lonardi *et al*., 2019; Munoz‐Amatriain *et al*., 2017; Spriggs *et al*., 2018; Yao *et al*., 2016), we believe that the broad application of this cowpea transformation and editing system will have an immediate and far‐reaching impact on cowpea research that will improve cowpea productivity.

## EXPERIMENTAL PROCEDURES

### *Agrobacterium* strains and vectors

Two *Agrobacterium* strains, the auxotrophic strain LBA4404 Thy‐ and AGL1, were used in this study. *Agrobacterium* auxotrophic strain LBA4404 Thy‐ was used with the ternary vector transformation system for cowpea IT86D‐1010 transformation. The ternary vector system contains the T‐DNA binary vector and the optimized pVIR accessory (pPHP71539) plasmid as previously described by Che *et al*. (Che *et al*., 2018) and Anand *et al*. (Anand *et al*., 2018). The T‐DNA binary plasmid pPHP86170 (Figure S1(a)) contains the PUC ORI, the *NPTIII* bacterial selectable marker, the *TagRFP* reporter gene and *spcN* (Anada *et al*., 2017) as the plant selectable marker gene. The binary plasmid pPHP94518 (Figure S1(b)) contains the PVS1 ORI, the SPEC bacterial selectable marker, the *Ds‐RED* reporter gene and *NPTII* as the plant selectable marker gene. The production binary plasmid pPHP92782 (Figure S1(c)) contains the PVS1 ORI, the *NPTIII* bacterial selectable marker, the *Ds‐RED* reporter gene and *spcN* (Anada *et al*., 2017) as the plant selectable marker gene. The ternary design was assembled by first mobilizing the accessory plasmid pPHP71539 in the *Agrobacterium* auxotrophic strain LBA4404 Thy‐ and selected on media supplemented with gentamycin (25 mg L^−1^). Subsequently, the binary constructs were electroporated into *Agrobacterium* strain LBA4404 Thy‐ containing the accessory plasmid. Recombinant colonies were selected on media supplemented with gentamycin plus either kanamycin for pPHP86170 and pPHP92782 or SPEC for pPHP94518 (Figure S1). All constructs were then subjected to next‐generation sequencing for sequence confirmation before conducting transformation experiments.

AGL1 carrying RC2717, a modified pAGM4673 vector (Figure S1(d)), was used for testing transformation on different cowpea germplasms other than IT86D‐1010. The T‐DNA region of the binary vector RC2717 contained a soybean‐codon‐optimized *aadA1* gene (Weber *et al*., 2011) as a selectable marker and a reporter cassette in which a *TdTomato* reporter cassette flanked by two *loxP* sites was placed in a reversed orientation between a soybean‐codon‐optimized *ZsGreen* gene and the Arabidopsis *Ubiquitin 10* promoter (Figure S1(d)). The *TdTomato* gene provided a visible fluorescence marker to identify transgenic shoots after transformation. With the use of the freeze‐thaw method (Chen *et al*., 1994), the binary vector was introduced into AGL1 and the recombinant colonies were selected on medium containing 100 mg L^−1^ kanamycin.

CRISPR/Cas gene editing was achieved using the LBA4404 Thy‐ *Agrobacterium* strain and the pPHP71539 pVIR accessory system described by Che *et al*. (Che *et al*., 2018) and Anand *et al*. (Anand *et al*., 2018) The *Streptococcus pyogenes* Cas9 (spCas9) and sgRNA gene editing machinery and the *spcN* selectable marker were expressed on a T‐DNA expressing binary vector (pPHP96249) (Figure S1(e)). The spCas9 is driven by a soybean elongation factor *EF1A2* promoter (Li *et al*., 2015) and the sgRNA cassette fused with CRISPR RNA (crRNA) and *trans*‐activating crRNA is driven by a cowpea U6 promoter (*Vu‐U6.1*) (Juranic *et al*., 2020). The N20 region of the crRNA NA hybridization region represents the RNA sequence used to target the genomic sequence upstream of the DNA triplet ‘NGG’ in the *VU‐SPO11* gene (Figure S8) that is recognized by the spCas9 enzyme. The guide RNA Vu‐SPO11‐CR2 (Figure S8) is the same as *SPO11‐1sg3* as described (Juranic *et al*., 2020).

### Plant materials and growth conditions

Cowpea varieties IT86D‐1010, PI 527675, PI 580227, PI 582835, PI583259, TVu 8670, TVu 3562, TVu 9693 and TVu 79, originally obtained from the U.S. NPGS (https://www.ars‐grin.gov/npgs/), were used for this study. Mexican cowpea accessions TPC1‐001 and MRS‐001 were collected from local farming communities in the Mexican states Tabasco and Morelos, respectively; the common bean varieties *black* bean (CBB‐001) and *pinto* bean (CBP‐001) were obtained from local producers in the Mexican state Guanajuato. Those varieties were maintained in the greenhouse to collect mature seeds for EA explant isolation.

### Cowpea transformation procedure

The main steps of cowpea transformation mediated by *Agrobacterium* are illustrated in Figure S2. The detailed protocol is described below. Unless otherwise specified, all the chemicals used for medium preparation were obtained from Sigma‐Aldrich.

#### Agrobacterium preparation

1. Master plate preparation: Streak *Agrobacterium* from glycerol stock on the master plate medium (Table S1) containing different antibiotics based on the *Agrobacterium* strains and the constructs that bacterium carries to make master plates. Incubate the master plates at 28°C for 3–4 days. The master plates can be kept in the refrigerator to make working plates and last for a month.
2. Working plate preparation: Streak a working plate on the working plate medium (Table S2) using a loop of bacteria from the master plate prepared above and incubate the working plate at 28°C overnight or for 20 h for LBA4404 Thy‐ and AGL1, respectively.
3. Inoculum preparation: Collect five to seven full loops of bacteria from the working plate using a sterile loop, suspend bacteria in 30 ml infection medium (IM) (Table S3) with acetosyringone (1 m stock in DMSO protected from light, final concentration, 200 µm) and dithiothreitol (DTT, 1 m stock, final concentration, 1 mm) freshly added in a sterile 50‐ml centrifuge tube. Adjust OD to 0.5.

#### Cowpea EA explant preparation

1. Seed sterilization: Cowpea seeds are surface‐sterilized using chlorine gas made by mixing in 3.5 ml of 12 m HCl and 100 ml bleach (5.25% sodium hypochlorite) for 16 h.
2. Seed pre‐treatment: Soak sterilized cowpea IT89D‐1010 seeds in bean germination medium (BGM) (Table S4) with approximately 45 ml of water for approximately 16 h. For other cowpea varieties transformed with AGL1, 30 ml 0MS medium (Table S8) is used to replace BGM.
3. EA explant isolation: Isolate embryonic axis (EA) explants with plumules by removing the seed coats and cotyledons and placing them into sterile water in a Petri dish until infection.

#### Agrobacterium inoculation

1. Infection: Remove water from the Petri dish (as much as possible), and add 15 ml inoculum and 50 µl sterile Poloxamer 188 10% solution. Wrap the plate with parafilm and sonicate (VWR Motel 50T or FS30H, Fisher Scientific, 120 V, 1 A) for 3 sec. After sonication, add an additional 10 ml of inoculum (total 25 ml in Petri dish) to the mix and gently shake on a shaker at approximately 60 rpm for 1.5 h at room temperature.
2. Co‐cultivation: Remove bacteria and transfer EAs to filter paper (VWR Cat No. 28320‐020) blotted with 700 µl IM in a 100×25 mm Petri dish. Thirty EAs can be piled up on the paper (two to four piles per plate). Seal plates with micropore tape and keep plates at 21°C, 45% relative humidity, 4.0 μmol m^−2^ s^−1^ light intensity for 2 days.

#### Cowpea regeneration

1. Regeneration with selection: Insert the roots of EAs vertically into SIM (Table S5) with cot‐node and SAM above the medium. Incubate the EAs on SIM at 26°C under 24 h light conditions. Remove the SAM and plumules together by cutting through the middle of the epicotyl using a pair of surgical scissors after 4–5 days of culturing on SIM to promote axillary shoot formation at the cot‐node region.
2. Rooting: After 3–5 weeks of regeneration, harvest shoots larger than 3 cm by cutting at the base of the shoot, and place into RIM (Table S6).
3. Shoot elongation (optional): If shoots do not reach 3 cm after 3–5 weeks of regeneration, they should be transferred to shoot elongation medium (SEM) (Table S7) for 2–4 weeks before transfer to RIM.

### Microscopy and imaging

Images were taken using a dissecting Leica M165 FC stereo‐epifluorescence microscope, with RFP and Ds‐RED filters for the detection of fluorescence, using the PLANAPO 1.0× objective, 0.63× zoom and Leica Application Suite V4.7 acquisition software. The autofluorescence of the wild‐type regenerated cowpea was evaluated using the same system. For testing transformation on the eight additional cowpea germplasm lines from NPGS, transgenic shoots expressing TdTomato were monitored with a Stemi SVII dissection stereoscope equipped with an HBO illuminator (Carl Zeiss, Thornwood, NY) and a Ds‐RED filter (excitation: 545/25 nm, emission: 605/70 nm, Chroma Technology, Bellows Falls, VT). Images were taken with an AxioCam camera (Carl Zeiss, Oberkochen, Germany) and AxioVision LE64 software and composed using Photoshop CC (Adobe, San Jose, CA).

### Evaluation of transgenic plants

The integrated copy number of the T‐DNA of the binary vector in the transgenic plants was determined by a series of quantitative PCR (qPCR) analyses based on the method previously described by Wu *et al*. (2014). In this study, an endogenous control qPCR assay (*LBS*) (Table S10) was developed using the house‐keeping gene *3‐isopropylmalate dehydrogenase* (Vigun05g298700), which is involved in the leucine biosynthetic pathway in cowpea (Misra *et al*., 2017). Five qPCR assays (*PINII_TERM*, *PSJ*, *spcN_SO*, *CTP* and *UBQ14_TERM*) for pPHP86170 (Figure S1(a) and Table S10) and five qPCR assays (*Ds‐RED*, *spcN_SO*, *PSJ*, *PSA2* and *PSB1*) for pPHP92782 (Figure S1(c) and Table S10) were developed to determine the T‐DNA copy number by normalizing with the endogenous control *LBS* assay. Two qPCR assays, *PSA2* and *PSB1*, were specially designed for pPHP92782 just within the right and left border regions to determine not only the copy number, but also intactness of the integrated T‐DNA (Figure S1(c)).

Outside the border integration sites, PCR backbone‐specific assays were developed to check for any border read‐through (Wu *et al*., 2014). The presence or absence of *Agrobacterium* vector backbone integration of the binary vector was detected based on screening for sequences from three regions outside of the T‐DNA integration sites for each vector, such as *SPC*, *LEFTBORDER* and *NPTIII* for plasmid pPHP92782 and *HYG*, *VIRG* and *HYGROMYCIN* for plasmid pPHP86170 (Figure S1(a) and Table S10).

Stable T‐DNA integration was confirmed by copy number determination using genomic DNA extracted from the putative T0 transgenic events. The T0 transgenic plants carrying a single copy of the intact T‐DNA integrations without vector backbone for all assays described were defined as quality events (Che *et al*., 2018; Zhi *et al*., 2015). The percentage of quality events was divided by the total number of events analyzed to calculate the quality event frequency. Only quality events were advanced to the greenhouse for the next generation. The zygosity of the T1 plants was established by determining the copy number of the T‐DNA for all the event quality assays (Figure S1 and Table S10). Chi‐square analysis was performed to determine whether the difference between the observed and the expected ratio was statistically significant.

### Amplicon deep sequencing

CRISPR/Cas edits were characterized by amplicon sequencing from DNA extracted from a single fresh leaf punch from each plant as per the manufacturer recommendations via the sbeadex^TM^ tissue extraction system (LGC Limited, UK). DNA concentration was adjusted to 10 ng µl^−1^ and 20‐cycle target region PCR was performed on 50 ng of genomic DNA with Phusion^TM^ Flash 2× master mix (Thermo Scientific, Waltham, MA) as per the manufacturer’s recommendations. Primary PCR product (5 µl) was used for 20‐cycle secondary amplification containing primers to attach individual sample indices and sequencing components, again with Phusion^TM^ Flash 2× master mix. Paired‐end sequencing was performed on an Illumina MiSeq®, with 150 cycles per read following the Illumina standard operating procedure. Sequence reads were aligned to the wild‐type reference sequence via Bowtie2. The allele return threshold was set to 5%. The primers used to amplify *VU‐SPO11* genomic loci are listed in Table S11. Edited sequences are reported in Table S12.

## AUTHOR CONTRIBUTIONS

PC, SC, MS, MA and TJ conceptualized the methods. PC, SC, MS, ZZ, JV, PO, MA and TJ designed research, analyzed the data and wrote the paper. AS conducted cowpea transformation and collected the data for IT86D‐1010. ZZ, YG and KM conducted transformation and genotyping for other cowpea genotypes collected from NPGS. JO and DO developed event quality assays. MT conducted regeneration experiments for Mexican cowpeas and beans.

## CONFLICT OF INTEREST

The authors have no conflict of interest to declare.

## Supporting information


**Figure S1.** Schematic representation of the constructs used in this study.
**Figure S2.** Diagram of the cowpea EA‐based *Agrobacterium*‐mediated transformation process. The detailed transformation procedure is described in the Experimental Procedures section.
**Figure S3.** Dry mature seeds of selected accessions of cowpea and common bean.
**Figure S4.** Shoot organogenesis of selected accessions of cowpea and common bean.
**Figure S5.** Development of chimeric events using the *CTP‐NPTII*/G418 selection system. (a) Bright field image. (b) Fluorescence image under RFP filter.
**Figure S6.** Transgene segregation in the progeny. (a) Mature wild‐type cowpea IT86D‐1010 seeds. (b) Segregated T1 seeds in the IT86D‐1010 background harvested from T0 plants containing *proGM‐EF1A2:Ds‐RED* as visual marker.
**Figure S7.** Formation of transgenic shoots expressing *TdTomato* on the EA explants of nine cowpea germplasm lines after 14‐day culture on SIM. Bar = 1 mm.
**Figure S8.** Diagram of the DNA sequence of the *VU‐SPO11* target site in exon 3.
**Table S1.** Master plate medium.
**Table S2.** Working plate medium
**Table S3.** Infection medium (IM).
**Table S4.** Bean germination medium (BGM).
**Table S5.** Shoot induction medium (SIM).
**Table S6.** Root induction medium (RIM).
**Table S7.** Shoot elongation medium (SEM).
**Table S8.** 0MS.
**Table S9.** Shoot organogenesis of selected accessions of cowpea and common bean.
**Table S10.** Primers used for event quality assays.
**Table S11.** Primers for the CRISPR/Cas target site.
**Table S12.** Sequence changes in Cas9‐edited plants.

## Data Availability

All relevant data supporting the findings of this work are available within the manuscript and the supporting materials. Novel biological materials described in this publication may be available to the academic community and other not‐for‐profit institutions solely for non‐commercial research purposes upon acceptance and signing of a material transfer agreement between the author’s institution and the requester. In some cases, such materials may originally contain genetic elements described in the manuscript that were obtained from a third party (e.g., *TagRFP*, *Ds‐RED* and *Cas9*), and the authors may not be able to provide materials including third‐party genetic elements to the requester because of certain third‐party contractual restrictions placed on the author’s institution. In such cases, the requester will be required to obtain such materials directly from the third party. The authors and the authors’ institution do not grant any express or implied permission(s) to the requester to make, use, sell, offer for sale or import third‐party proprietary materials. Obtaining any such permission(s) will be the sole responsibility of the requester. In order to protect Corteva Agriscience^TM^ proprietary germplasm, such germplasm will not be made available except at the discretion of Corteva Agriscience^TM^ and then only in accordance with all applicable governmental regulations.
