# Supplementary material for: Developing a rapid and highly efficient cowpea regeneration, transformation and genome editing system using embryonic axis explants
Source: Plant J. 2021 Mar 16;106(3):817–30. doi: 10.1111/tpj.15202 (PMC8252785; doi:10.1111/tpj.15202)
Supplement: Supplementary file 1 — Figure S1. Schematic representation of the constructs used in this study. Figure S2. Diagram of the cowpea EA‐based Agrobacterium‐mediated transformation process. The detailed transformation procedure is described in the Experimental Procedures section. Figure S3. Dry mature seeds of selected accessions of cowpea and common bean. Figure S4. Shoot organogenesis of selected accessions of cowpea and common bean. Figure S5. Development of chimeric events using the CTP‐NPTII/G418 selection system. (a) Bright field image. (b) Fluorescence image under RFP filter. Figure S6. Transgene segregation in the progeny. (a) Mature wild‐type cowpea IT86D‐1010 seeds. (b) Segregated T1 seeds in the IT86D‐1010 background harvested from T0 plants containing proGM‐EF1A2:Ds‐RED as visual marker. Figure S7. Formation of transgenic shoots expressing TdTomato on the EA explants of nine cowpea germplasm lines after 14‐day culture on SIM. Bar = 1 mm. Figure S8. Diagram of the DNA sequence of the VU‐SPO11 target site in exon 3. Table S1. Master plate medium. Table S2. Working plate medium Table S3. Infection medium (IM). Table S4. Bean germination medium (BGM). Table S5. Shoot induction medium (SIM). Table S6. Root induction medium (RIM). Table S7. Shoot elongation medium (SEM). Table S8. 0MS. Table S9. Shoot organogenesis of selected accessions of cowpea and common bean. Table S10. Primers used for event quality assays. Table S11. Primers for the CRISPR/Cas target site. Table S12. Sequence changes in Cas9‐edited plants. [file TPJ-106-817-s001.pdf]

Supporting information

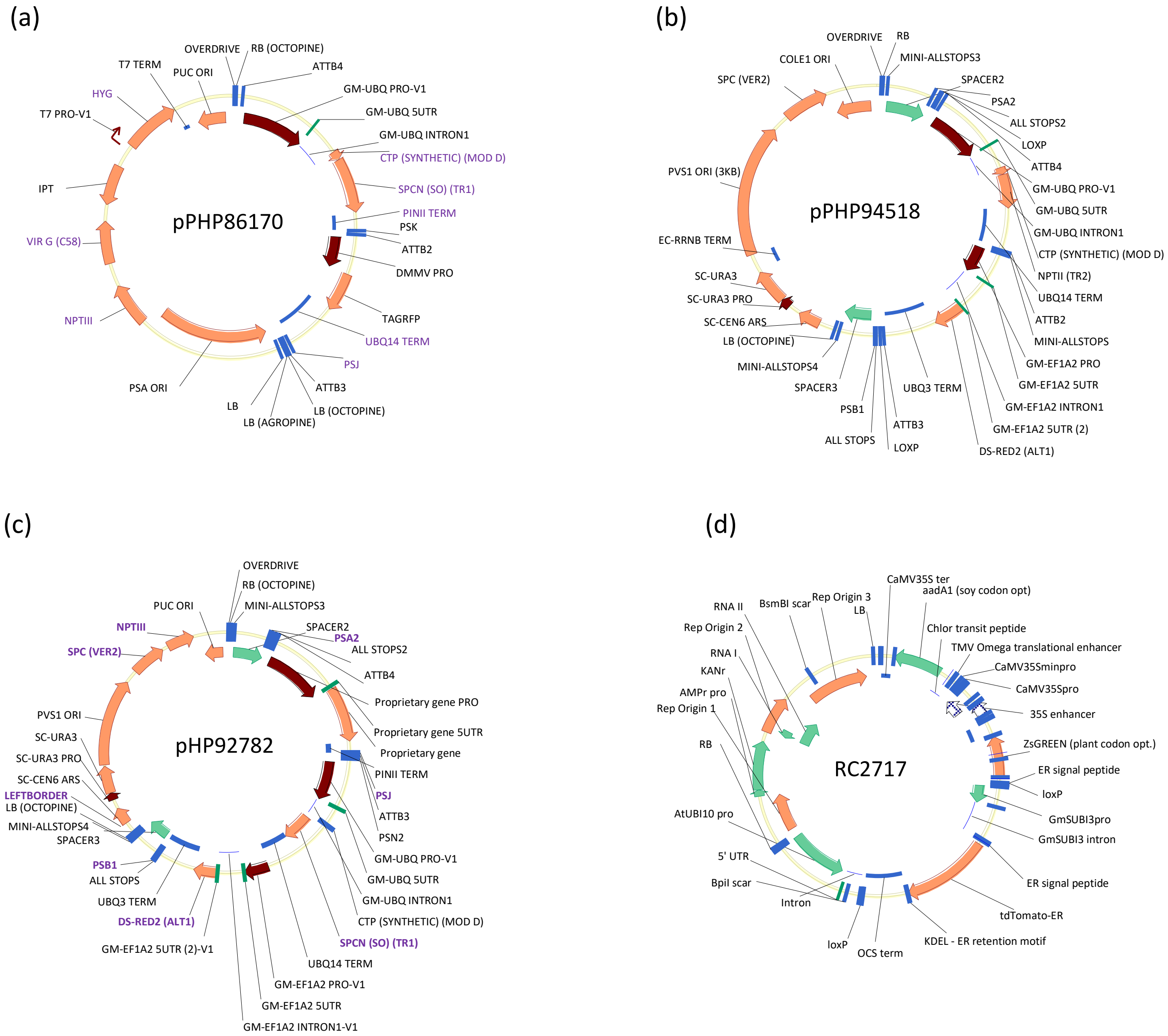

(e)

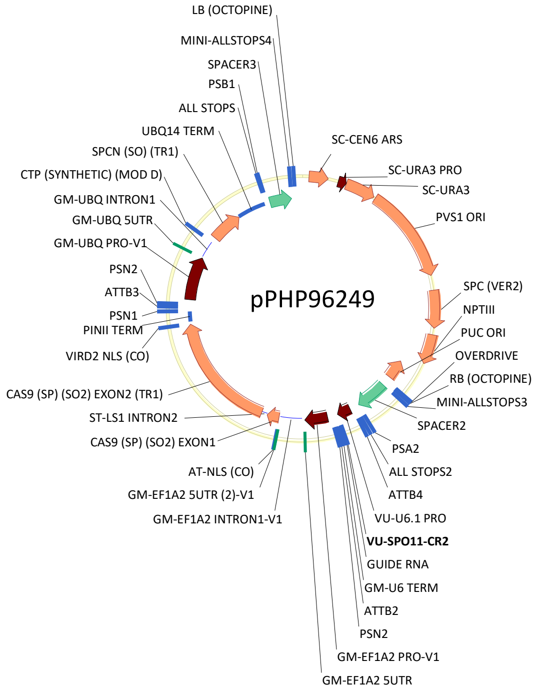

Figure S1. Schematic representation of the molecular components of constructs used in this study. (a) pPHP86170, (b) pPHP84518, (c) pPHP92782 and (e) pPHP96249 were transformed with ternary vector system using pPHP71539 as helper in *Agrobacterium* strain LBA4404 Thy-. The genes and elements highlighted in purple are covered by event quality assays (Table S10). (d) RC2717 is a modified pCAMBIA vector transformed into *Agrobacterium* AGL1 strain. (e) Gene editing construct for *Vu-SPO11.*

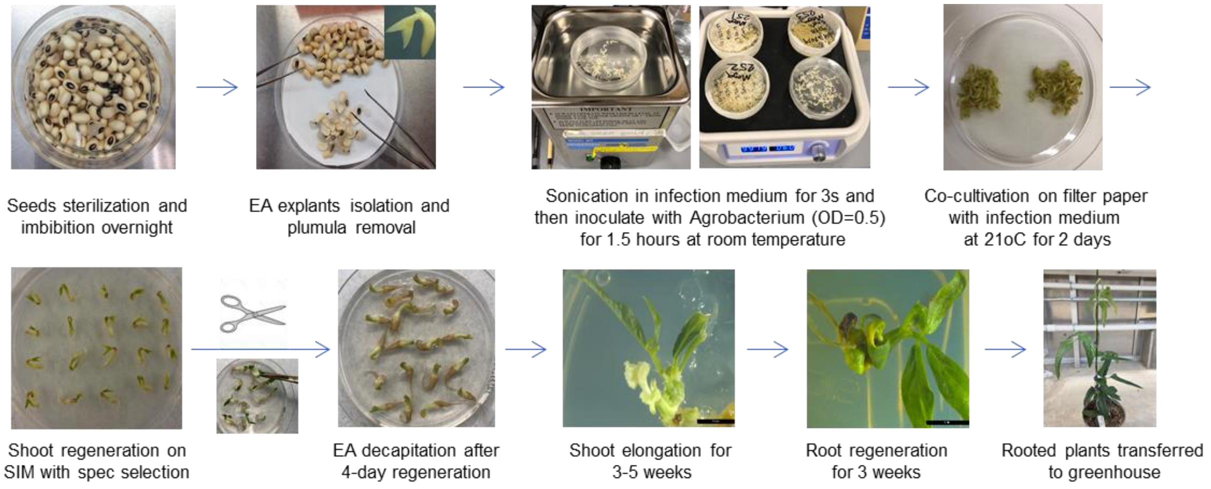

Figure S2. Diagram of the cowpea EA-based *Agrobacterium*-mediated transformation process.

The detailed transformation procedure is described in Materials and Methods.

---Internal Use---

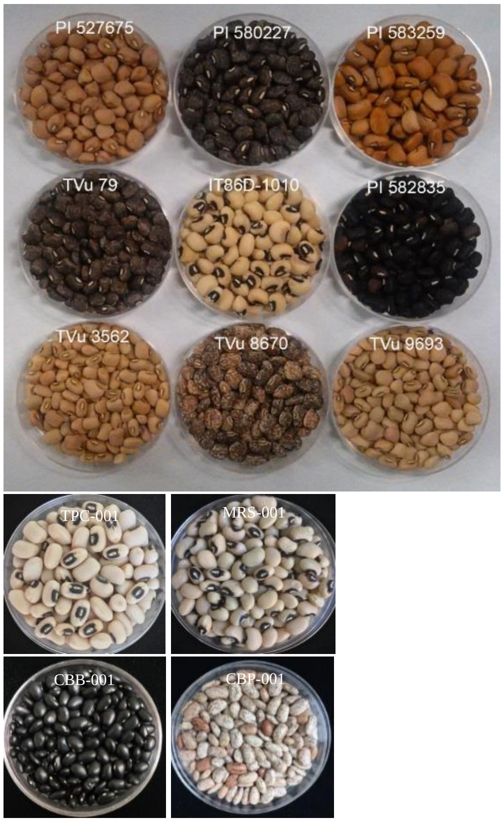

Figure S3. Dry mature seeds of selected accessions of cowpea and common bean.

---Internal Use---

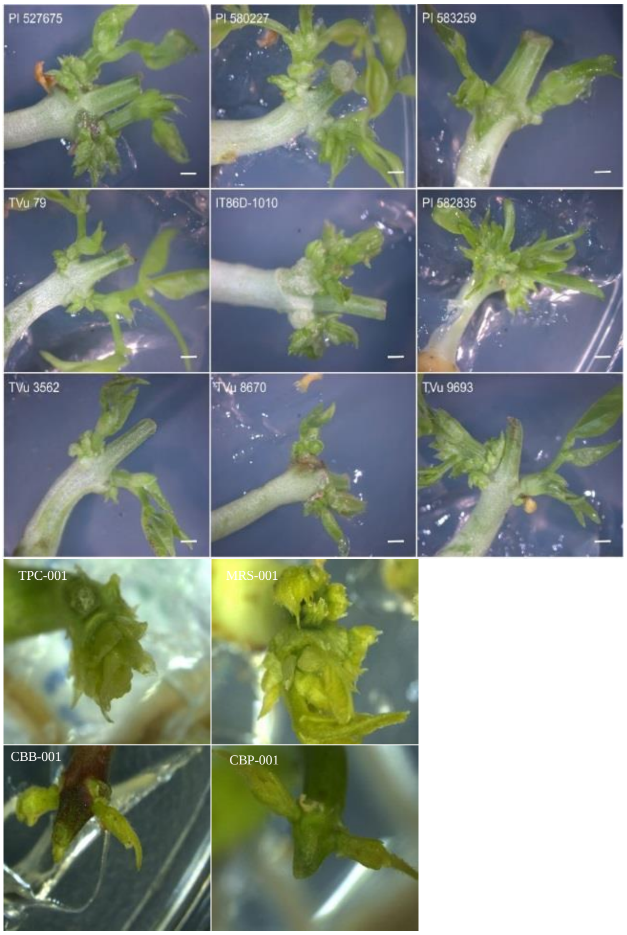

Figure S4. Shoot organogenesis of selected accessions of cowpea and common bean.

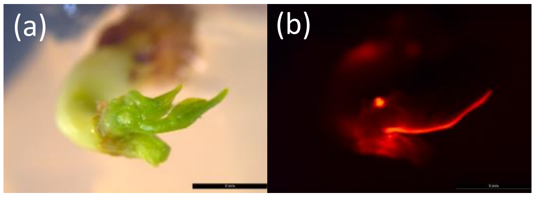

Figure S5. Development of chimeric event using *CTP-NPTII*/G418 selection system. (a) Bright field image. (b) Fluorescence image under RFP filter.

---Internal Use---

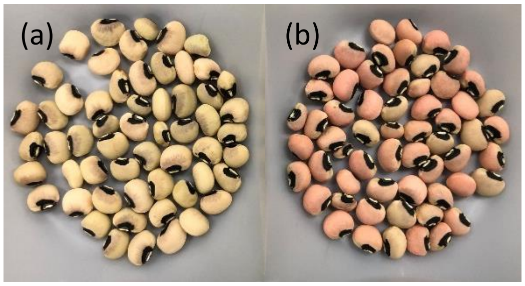

Figure S6. Transgene segregation in the progeny. (a) Mature wild-type cowpea IT86D-1010 seeds. (b) Segregated T1 seeds (Event ID 125739950) in IT86D-1010 background harvested from T0 plant containing the *proGM-EF1A2:Ds-RED* as visual marker.

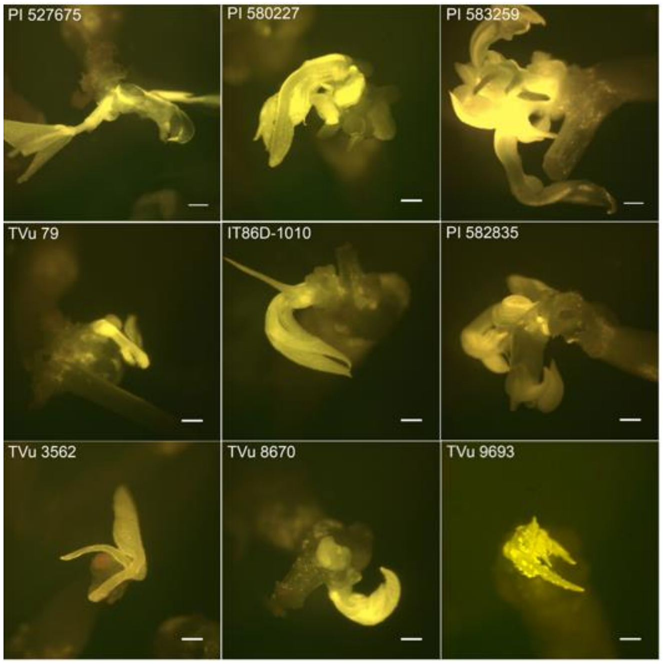

Figure S7. Formation of transgenic shoots expressing *TdTomato* on the EA explants of nine cowpea germplasm lines after 14-d culture on SIM. bar = 1 mm.

---Internal Use---

Vu-SPO11-CR2

5'-CTTTTGTGCCTCCGATTTGCCGTGCGGAAAGGAAGCTCTCACACTCCGCACGAAATCCCGCGCGCACAGACTAG-3'

3'-GAAAACACGGAGGCTAAACGGCACGCCTTTCCTTCGAGAGTGTGAGGCGTGCTTTAGGGCGCGCGTGTCTGATC-5'

Figure S8. Diagram of the DNA sequence of *VU-SPO11* target site in Exon 3. The three-nucleotide sequence highlighted in grey indicate the corresponding PAM motif recognized by the Cas9 protein. The sgRNA target site is underlined.

Table S1 Master plate medium

Glucose	5 g/l
Bacto agar	15 g/l
Ferrous sulfate heptahydrate	2.5 mg/l
Potassium phosphate dibasic	3 g/l
Sodium phosphate, monobasic	1 g/l
Ammonium chloride	1 g/l
Magnesium sulfate heptahydrate	0.3 g/l
Potassium chloride	0.15 g/l
Calcium chloride dihydrate	11.4 mg/l
Thymidine (only for LBA4404 THY- strain)	50 mg/l
Antibiotics*	

*****100 mg/l kanamycin, 100 mg/l carbenicillin and 25 mg/l rifampicin were used for AGL1 carrying RC2717.
50mg/l gentamycin and 50 mg/l kanamycin were used for LBA4404 Thy- carrying pPHP86170/pPHP71539.
50mg/l gentamycin and 50 mg/l spectinomycin were used for LBA4404 Thy- carrying pPHP94518/pPHP71539 and pPHP92782/ pPHP71539.

Table S2 Working plate medium

Yeast extract (BD DIFCO)	5 g/l
Peptone	10 g/l
Sodium chloride	5 g/l
Bacto agar	15 g/l
Thymidine (only for LBA4404 THY- strain)	50 mg/l
Antibiotics*	

*****100 mg/l kanamycin, 100 mg/l carbenicillin and 25 mg/l rifampicin were used for AGL1 carrying RC2717.
50mg/l gentamycin and 50 mg/l kanamycin were used for LBA4404 Thy- carrying pPHP86170/pPHP71539.
50mg/l gentamycin and 50 mg/l spectinomycin were used for LBA4404 Thy- carrying pPHP94518/pPHP71539 and pPHP92782/ pPHP71539.

---Internal Use---

Table S3 Infection medium (IM)

MS salt	1 x
MS vitamins	1 x
MES	20 Mm (3.9 g/l)
Sucrose (w/v)	30 g/l
pH	5.4
BAP	0.5 mg/l
Kinetin	0.5 mg/l
GA3	0.25 mg/l
Acetosyringone	Add fresh
L-cysteine	400 mg/l
BCDA (bathocuproinedisulfonic acid disodium salt) (filter sterilized stock 125mM)	800 µL stock
Thymidine (only for LBA4404 THY-)	50 mg/l
Polyvinylpyrrolidone (PVP40)	1 mg/l
Acetosyringone (stock 1M; final 200 µM)	0.2 mL
Dithiothrietol (DTT, stock 1M, filter sterilized aliquot and stored at -80°c; final 1Mm)	1 mL

Table S4 Bean germination medium (BGM)

Sucrose	25 g/l
Thiamine Hydrochloride	1.34 mg/l
Nicotinic acid	0.5 mg/l
Pyridoxine Hydrochloride	0.82 mg/l
EDTA disodium	3.348 mg/l
Ferrous sulfate heptahydrate	2.502 mg/l
Boric acid	1.86 mg/l
Manganese sulfate, monohydrate	5.07 mg/l
Zinc sulfate, heptahydrate	2.58 mg/l
Potassium iodide	0.249 mg/l
Sodium molybdate dihydrate	0.216 mg/l
Cupric sulfate pentahydrate	0.00075 mg/l
Cobalt chloride hexahydrate	0.00075 mg/l
Calcium chloride dihydrate	0.176 g/l
Potassium nitrate	0.505 g/l
Ammonium nitrate	0.24 g/l
Potassium phosphate monobasic anhydrous	0.027 g/l
Magnesium sulfate heptahydrate	0.493 g/l
TC agar (phytotechnology A296)	6 g/l

---Internal Use---

Table S5 Shoot induction medium (SIM)

MS salt	1 x
MS vitamins	1 x
MES	3 Mm (0.59 g/l)
Sucrose (w/v)	30 g/l
Agar, DIFCO (w/v)	8 g/l
pH	5.6
BAP	0.5 mg/l
Kinetin	0.5 mg/l
Antibiotic selection*	
Polyvinylpyrrolidone	1 mg/l
Silver nitrate	2 mg/l

*25 mg/l spectinomycin and 15 mg/l meropenem was used for EA explants which transformation was carried out through LBA4404 Thy- strain-mediated transformation.

50 mg/l spectinomycin and 15 mg/l meropenem was used for EA explants which transformation was carried out through AGL1 strain-mediated transformation for the first 2 weeks culture and 50 mg/l spectinomycin and 30 mg/l meropenem after the first 2 weeks culture.

Table S6 Rooting induction medium (RIM)

MS salt	1 x
MS vitamins	1 x
MES	3 Mm (0.59 g/l)
Sucrose (w/v)	30 g/l
Agar, DIFCO (w/v)	8 g/l
pH	5.6
IBA	0.1 mg/l
Antibiotic selection*	
Polyvinylpyrrolidone	1 mg/l
Sliver nitrate	2 mg/l

*50 mg/l spectinomycin and 30 mg/l meropenem used for transgenic shoots which transformation were carried out through AGL1 strain-mediated transformation after the first 2 weeks culture.

Table S7 Shoot elongation medium (SEM)

MS salts	1 x
MS vitamins	1 x
MES	3 mM (0.59 g/l)
Sucrose (w/v)	30 g/l
Agar (w/v)	8 g/l
pH	5.6
GA	0.5 mg/l
Kinetin	0.1 mg/l
Asparagine	50 mg/l
Antibiotic selection*	

*25 mg/l spectinomycin and 15 mg/l meropenem used for transgenic shoots which transformation was carried through LBA4404 Thy- strain-mediated transformation.

50 mg/l spectinomycin and 30 mg/l meropenem used for transgenic shoots which transformation was carried out by AGL1 strain-mediated transformation.

---Internal Use---

Table S8 0MS

MS salts	1 x
MS vitamins	1 x
MES	3 Mm (0.59 g/l)
Sucrose (w/v)	30 g/l
Agar, DIFCO (w/v)	8 g/l
pH	5.6

Table S9 Shoot organogenesis of selected accessions of cowpea and common bean

Germplasm accessions	# of EAs	# of EAs with single shoot (%)	# of EAs with multiple shoots (%)	% Explants with shoots
Cowpea				
IT86D-1010	36	0 (0)	28 (78)	78
PI 527675	36	0 (0)	29 (81)	81
PI 580227	36	2 (6)	26 (72)	78
PI 582835	29	1 (3)	22 (76)	79
PI 583259	36	3 (8)	17 (47)	56
TVu 8670	36	2 (6)	22 (61)	67
TVu 3562	37	4 (11)	21 (57)	68
TVu 9693	36	0 (0)	22 (61)	61
TVu 79	36	1 (3)	28 (78)	81
Mexican cowpea				
TPC-001	50	6 (12)	12 (24)	36
MRS-001	50	5 (10)	14 (28)	38
Common bean				
CBB-001	50	4 (8)	11 (22)	30
CBP-001	50	6 (12)	8 (16)	30

Table S10 Primers used for event quality assay

Event quality assay	Assay type	Forward primer	Reverse primer
LBS	Endogenous control	CACATACCTCCAGTGAGTTCCCTTA	TCGAAGCATCTACTAACTACAGAAGAATTAA
Ds-RED	Copy number	AAGTCCATCTACATGGCCAAGAA	TGGGAGGTGATGTCCAGCTT
PSJ	Copy number	GCTAGTAGACGCTGCTAGTGACTAAGG	GCACTTAGGAGCGAAGACTAACG
SPCN	Copy number	CTGCCCGCAATGCTCTTT	ATTACCACTGGACCGTCACAGA
PSA2	Copy number and full insert intactness	CATGAAGCGCTCACGGTTACTAT	TCGTACGCTACTGCCACCAA
PSB1	Copy number and full insert intactness	TGATTCCGATGACTTCGTAGGTT	GCTAATCGTAAGTGACGCTTGGA
CTP	Copy number	TGGCTGCAACTACTCTTACATCTG	TGTAAGTTGAAAGGAGCACTTGGT
UBQ14_TERM	Copy number	CAGAACCCAGAATCCCTTCATATC	TGACGGCTGGGACTTCTTTG
HYGROMYCIN	Vector backbone	CAGCGAGAGCCTGACCTATTG	CAGCGAGAGCCTGACCTATTG
VIRG	Vector backbone	TGCTCCGAGACGGTCGAT	CAGGCAGGTCTTGCAACGT
SPC	Vector backbone	GCGCTGCCATTCTCCAAAT	ATCATTCCGTGGCGTTATCC
LEFTBORDER	Vector backbone	GATCTCGCGGAGGGTAGCA	CGAGGGAGATGATATTTGATCACA
NPTIII	Vector backbone	CCGATGTGGATTGCGAAAA	GCTCGCGCGGATCTTTAA

---Internal Use---

Table S11 Primers for CRISPR/Cas target site

Gene Target	Forward Primer	Reverse Primer
VU-SPO11-CR2	TCCAACTAATCACCGTTTCACC	GCACAGACTAGGTCACTCTCCTTTT

Table S12 Sequence changes from Cas9 edited plants

Event ID	Allele Change	Δ	Allele read percentage
1	GC[-G]GAA	-1bp	94%
2	GCGGAA	Wildtype	86%
3	GCGGAA	Wildtype	88%
4	C[-CGTG]CGGAA	-4bp	42%
4	GC[+G]GGAA	+1bp	37%
4	GCGGAA	Wildtype	16%
5	GCGGAA	Wildtype	66%
5	GC[-G]GAA	-1bp	9%
6	GCGGAA	Wildtype	57%
6	GC[-G]GAA	-1bp	18%
7	GCGGAA	Wildtype	48%
7	GC[-G]GAA	-1bp	16%
7	GC[+G]GGAA	+1bp	6%
8	GCGGAA	Wildtype	45%
8	GC[-G]GAA	-1bp	15%
9	G[-CGGAAAGGAAGCTCTCACACTC]	-22b	52%
9	GC[+G]GGAA	+1bp	42%
10	GCGGAA	Wildtype	55%
10	GC[+G]GGAA	+1bp	38%
11	GCGGAA	Wildtype	88%
12	GC[-GGA]A	-3bp	40%
12	GC[+G]GGAA	+1bp	31%
12	GCGGAA	Wildtype	23%
13	GC[-G]GAA	-1bp	74%
13	GCGGAA	Wildtype	19%
14	[-GCG]GAA	-3bp	39%
14	[-GC]GGAA	-2bp	34%
14	GCGGAA	Wildtype	20%
15	[-GCG]GAA	-3bp	38%
15	[-GC]GGAA	-2bp	33%
15	GCGGAA	Wildtype	14%
16	GCGGAA	Wildtype	91%
17	GCGGAA	Wildtype	51%
17	GC[-G]GAA	-1bp	12%
18	GCGGAA	Wildtype	85%
19	GCGGAA	Wildtype	88%
20	GCGGAA	Wildtype	38%
20	GC[-G]GAA	-1bp	16%
21	GCGGAA	Wildtype	43%
21	GC[-G]GAA	-1bp	18%
21	GC[+G]GGAA	+1bp	8%
22	GCGGAA	Wildtype	49%
22	GC[-G]GAA	-1bp	11%
22	GC[-GGA]A	-3bp	10%
23	GCGGAA	Wildtype	51%
23	GC[-G]GAA	-1bp	12%
24	GCGGAA	Wildtype	92%
25	GCGGAA	Wildtype	76%
25	GC[-G]GAA	-1bp	7%
26	GCGGAA	Wildtype	57%
26	GC[+G]GGAA	+1bp	33%
27	GCGGAA	Wildtype	54%
27	GC[-G]GAA	-1bp	15%

---Internal Use---

28	GC[-G]GAA	-1bp	26%
28	GCGGAA	Wildtype	19%
28	GC[+G]GGAA	+1bp	8%
28	[-GC]GGAA	-2bp	8%
28	[-CGTG]CGGAA	-4bp	5%
29	GCGGAA	Wildtype	25%
29	GC[-G]GAA	-1bp	20%
29	GC[+G]GGAA	+1bp	6%
30	GCGGAA	Wildtype	85%
31	CTTTT[-GTGC]CT[-CCGATTTGCCGTGCGGAAAGGAA]G[-CTCTC]ACACTCCGGACGAAATCACGCGCGCACAGACTAG	Complex deletion	45%
31	GCGGAA	Wildtype	27%
31	GC[-G]GAA	-1bp	7%
32	GCGGAA	Wildtype	55%
32	GC[-G]GAA	-1bp	12%
33	GCGGAA	Wildtype	88%
34	GCGGAA	Wildtype	82%
35	GCGGAA	Wildtype	91%

---Internal Use---
